# Maculopathy and Dengue

**DOI:** 10.3201/eid1302.061105

**Published:** 2007-02

**Authors:** Augustinus Laude, Maciej Piotr Chlebicki, Brenda Ang, Timothy Barkham

**Affiliations:** * Tan Tock Seng Hospital, Singapore

**Keywords:** Retinal hemorrhage, dengue, ophthalmic complications, letter

**To the Editor:** We thank Su and Chee (*1*) for their interest in our article, “Retinal Hemorrhages in 4 Patients with Dengue Fever” (*2*). We reported the findings of this small case series to highlight the presence of retinal hemorrhage as a manifestation of ophthalmic complication in patients with dengue fever. We wanted to describe characteristic clinical features (such as association of onset of visual symptoms with resolution of fever and nadir of thrombocytopenia) and propose epidemiologic explanations for the sudden rise in the incidence of observed ocular complications of dengue fever in our population. Our article did not attempt to conclude that the retinal hemorrhages were responsible for the patients’ visual symptoms, as suggested by Su et al. In fact, we stated that in all 4 patients “fundoscopic examination showed macular hemorrhages and exudative maculopathy.”

The range of dengue-related ophthalmic complication is still being investigated, and we agree with Su and Chee that other ophthalmic manifestations may occur in patients with dengue fever. In a retrospective observational case series involving 22 eyes of 13 patients with visual impairment from dengue infection, carried out in our hospital, Chan et al. (*3*) found evidence of retinal hemorrhage, macular edema, cotton wool spots, retinal vasculitis, exudative retinal detachment, and anterior uveitis. Therefore, physicians and ophthalmologists should be aware of the possibilities of ophthalmic complications in the management of patients with dengue fever.
